# Development of an Online Tool for *Pasteurella multocida* Genotyping and Genotypes of *Pasteurella multocida* From Different Hosts

**DOI:** 10.3389/fvets.2021.771157

**Published:** 2021-12-17

**Authors:** Zhong Peng, Junyang Liu, Wan Liang, Fei Wang, Li Wang, Xueying Wang, Lin Hua, Huanchun Chen, Brenda A. Wilson, Jia Wang, Bin Wu

**Affiliations:** ^1^State Key Laboratory of Agricultural Microbiology, The Cooperative Innovation Center for Sustainable Pig Production, College of Animal Science and Veterinary Medicine, Huazhong Agricultural University, Wuhan, China; ^2^Hubei Key Laboratory of Agricultural Bioinformatics, College of Informatics, Huazhong Agricultural University, Wuhan, China; ^3^Key Laboratory of Prevention and Control Agents for Animal Bacteriosis (Ministry of Agriculture and Rural Affairs), Animal Husbandry and Veterinary Institute, Hubei Academy of Agricultural Sciences, Wuhan, China; ^4^Department of Microbiology, School of Molecular and Cellular Biology, University of Illinois at Urbana-Champaign, Urbana, IL, United States

**Keywords:** *Pasteurella multocida*, genotyping, whole genome sequence, PmGT, genotypes

## Abstract

*Pasteurella multocida* is a versatile zoonotic pathogen. Multiple systems have been applied to type *P. multocida* from different diseases in different hosts. Recently, we found that assigning *P. multocida* strains by combining their capsular, lipopolysaccharide, and MLST genotypes (marked as capsular: lipopolysaccharide: MLST genotype) could help address the biological characteristics of *P. multocida* circulation in different hosts. However, there is still lack of a rapid and efficient tool to diagnose *P. multocida* according to this system. Here, we developed an intelligent genotyping platform PmGT for *P. multocida* strains according to their whole genome sequences using the web 2.0 technologies. By using PmGT, we determined capsular genotypes, LPS genotypes, and MLST genotypes as well as the main virulence factor genes (VFGs) of *P. multocida* isolates from different host species based on their whole genome sequences published on NCBI. The results revealed a closer association between the genotypes and pasteurellosis rather than between genotypes and host species. With the advent of high-quality, inexpensive DNA sequencing, PmGT represents a more efficient tool for *P. multocida* diagnosis in both epidemiological studies and clinical settings.

## Introduction

Rapid and accurate diagnosis of sources of infections is critical for both medical and veterinary activities, and it is important for improved understanding of disease mechanisms and measures to control the illness (1). Microbial typing is an important link for the diagnosis of pathogens associated with diseases. The most widely used typing methods consist of serological typing systems and PCR-based molecular typing methods (2, 3). The establishment of discriminatory typing systems help in the understanding and control of pathogens, especially those with multiple serovars and/or genotypes from different environmental or host sources. Whole genome sequencing combined with the high-end computational technology is such an emerging approach for microbial diagnosis (4). Using the whole genome sequencing technologies, it is possible to determine the causative agent of infectious diseases rapidly and accurately, including newly emerged ones (5, 6). However, interpretation of the sequencing results to formulate a definitive diagnosis still requires technical experts with computational and bioinformatics skills. Therefore, a practical, automated platform that combines whole genome sequencing with computational technologies to provide diagnostic outcomes would be beneficial in advancing the field.

*Pasteurella multocida* is an important zoonotic pathogen and it can colonize and cause infections in a wide range of domestic and wild animals including food producing animals (e.g., poultry, pigs, beef, sheep) and companion animals (e.g., cats and dogs) as well as in humans (7–9). Animal diseases associated with *P. multocida* such as fowl cholera in poultry and other birds, progressive atrophic rhinitis and pneumonic pasteurellosis in pigs, haemorrhagic septicaemia and respiratory diseases in cattle and buffalos, leporine atrophic rhinitis and pneumonic pasteurellosis, are of great economic significance in agriculture (9). In humans, opportunistic infections of soft tissue, including wound dermonecrosis, respiratory disease with chronic pulmonary, urinary tract infection and bacteremic meningitis have also been reported (9). Most of these infections are associated with animal biting, scratching, kissing, and/or licking (10–12). In this regard, *P. multocida* represents a risk to public health. *P. multocida* strains from different hosts are serologically classified into five serogroups (A, B, D, E, F) (13–15) and/or 16 serovars (serovars 1 to 16) (16), according to their capsular and lipopolysaccharide (LPS) antigens, respectively. However, these two traditional serological typing methods require high-quantity antisera that are challenging to prepare, particularly for clinical use, such those methods are no longer widely used for large-scale epidemiological studies (7, 17).

In 2001, a multiplex PCR-based method was established to type the five serogroups into five capsular genotypes (A, B, D, E, F) (18), and in 2015, another multiplex PCR-based method was also developed to classified the 16 serovars into eight LPS genotypes (L1~L8) (19). In 2004 and 2010, two multilocus sequencing typing systems were also developed to genotype *P. multocida* strains (https://pubmlst.org/pmultocida/) from multiple mammalian hosts and birds, respectively (20, 21). In 2017, a virulence genotyping system based on the detection of different virulence factor gene (VFG) profiles was also reported for distinguishing *P. multocida* strains from different hosts (22). Compared to the traditional serological typing methods, these molecular DNA-based typing systems are indeed highly effective and accurate, and they are now widely used to determine the epidemiological and genetic characteristics of clinical isolates (23–27).

Despite of more than 135 years of research, differences on the molecular biological characteristics of *P. multocida* prevalence in different host species remain to be addressed. For example, *P. multocida* type A strains have been recovered from avian species, pigs, bovine species, and many other host species (8, 9), but little is known about differences on those type A isolates from different hosts. Recently, we developed a system to assign *P. multocida* strains from different host species by combining their capsular, LPS, and MLST genotypes (marked as capsular genotype: LPS genotype: MLST genotype), as well as determine the VFG profiles, which contributes to address the molecular biological characteristics of *P. multocida* prevalence in different host species (7, 23, 27). However, this strategy requires bioinformatics experts for data analysis and interpretation. Here, we report the development of an automated platform to type *P. multocida* strains from multiple hosts that combines the use of whole genome sequencing.

## Materials and Methods

### Bacterial Strains and Nucleotide Sequences

*P. multocida* strains used in this study include one isolate of bovine origin (strain HB01), one isolate of avian origin (strain HB02), and 50 isolates of porcine origin (strains HB03, HN04, HN05, HN06, HN07, HNA01~HNA22, HND01~HND21, HNF01 and HNF02) (Supplementary Table S1). All of these strains are from our laboratory collection, for which we have previously sequenced their whole genome sequences (27–30).

Nucleotide sequences specific for the determination of *P. multocida* strains (*KMT1*, 460 bp), and their the five capsular genotypes (A, 1044 bp; B, 760 bp; D, 657 bp; E, 511 bp; F, 851 bp); as well as their eight LPS genotypes (L1, 1307 bp; L2, 810 bp; L3, 474 bp; L4, 550 bp; L5, 1175 bp; L6, 668 bp; L7, 931 bp; L8, 255 bp) were extracted from the genome sequences of the different *P. multocida* strains according to the positions documented in previous publications (18, 19) and were deposited in GenBank under accession numbers MT570166, MN938443~MN938455 (Supplementary Text 1).

The nucleotide sequences of 23 types of virulence genes commonly detected in *P. multocida* epidemiological studies, including those encoding fimbriae and other adhesins (*ptfA, fimA, hsf-1, hsf-2, pfhA*, and *tadD*), toxin (*toxA*), iron acquisition proteins (*exbB, exbD, tonB, hgbA, hgbB, fur*, and *tbpA*), sialidases (*nanB* and *nanH*), hyaluronidase (*pmHAS*), outer membrane proteins (OMPs) (*ompA, ompH, oma87*, and *plpB*), and superoxide dismutase (*sodA* and *sodC*), were amplified from the genomic DNA of *P. multocida* HN06 and HB01 by PCR assays using the protocols documented elsewhere (23, 31). These nucleotide sequences were deposited in GenBank under accession numbers MT570167~ MT570189 (Supplementary Text 1).

The publicly available whole genome sequences of 262 *P. multocida* strains from bovine species [*n* = 106; including those recovered bovine haemorrhagic septicaemia cases (32)], avian species (*n* = 39), porcine species (*n* = 66), leporine species (*n* = 20), ovine species (*n* = 6), humans (*n* = 13), canines (*n* = 3), murine species (*n* = 2), horses (*n* = 2), cats (*n* = 2), alpacas (*n* = 2) and 1 synthetic DNA sequence in NCBI genome database were downloaded for use (Supplementary Table S1).

### System Implementation

The PmGT platform was integrated on a CentOS server, mainly providing two kinds of online services: genotyping tool, and data query and display. To establish the genotyping online service, we first used Apache (https://www.apache.org) as the web container. Then, we downloaded the BLAST package (ftp://ftp.ncbi.nlm.nih.gov/blast/executables/LATEST/) from NCBI, which was thereafter installed and configured on the web container. PHP was used as the server-side language and the browser-side script used jQuery, which is a fast, small, and feature-rich JavaScript library. The view pages were constructed with Hypertext Markup Language (HTML) and Cascading Style Sheets (CSS). For the target strain, the format of the sequence was first verified by the web user interface and then the sequence data was uploaded to the server through the PHP program which subsequently called the localized BLAST to align the uploaded sequence with the reference database. The nucleotide sequences specific for the determination of *P. multocida* strains, capsular genotypes, LPS genotypes, and the 23 types of virulence factor genes (VFGs) were packaged and used as the reference database for sequence alignment. Finally, the result was returned and displayed in the web page. In addition, if the user selected the option of “MLST genotyping,” the http request function “curl_setopt” in PHP was used to request PubMLST's RESTful interface (http://rest.pubmlst.org/db/pubmlst_Pmultocida_seqdef/sequence) and the function “curl_exec” was used to catch the response which thereafter was parsed to the result and displayed in the genotyping page.

### PCR Detection of Capsular Genotypes, LPS Genotypes, MLST Genotypes, and Virulence Genes of *P. multocida* Strains From Pigs

Capsular genotypes and LPS genotypes of *P. multocida* strains from our laboratory collection were determined using multiplex PCR-based assays, as documented elsewhere (18, 19). Profiles of 23 types of virulence genes mentioned above were determined by PCR assays, as described previously (23). Sequence types (STs) were determined according to the protocols described in *Pasteurella multocida* MLST database (https://pubmlst.org/organisms/pasteurella-multocida/multi-host).

### Data Availability

Nucleotide sequences specific for *P. multocida* and its capsular genotypes, LPS genotypes, as well as VFGs were publicly available in GenBank under accession numbers MN938443-MN938455 and MT570167~MT570189. The typing system developed in the present study is available at: http://vetinfo.hzau.edu.cn/PmGT.

## Results

### Development and Implementation of PmGT

**Figure 1:**
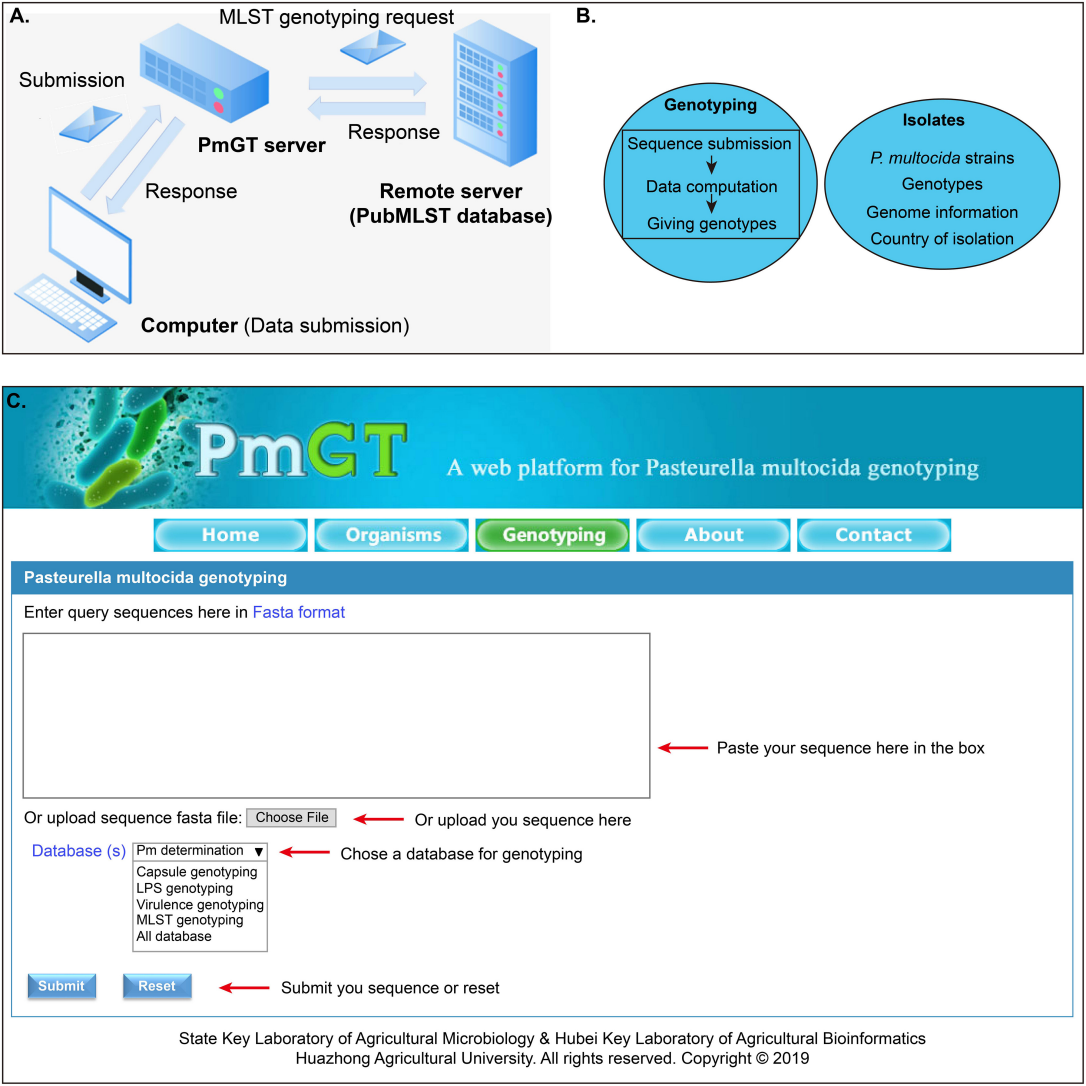
Development of the *P. multocida* genotyping and host prediction platform. **(A)** Flowchart showing the system design; **(B)** Main functions of the web platform; **(C)** Overview of the genotyping system of *P. multocida*.

The general process for genotyping is summarized as: when a query sequence is submitted via the web user interface, this sequence will be then submitted to the CentOS server via HTTP protocol. Thereafter, the sequence is evaluated by the PHP program, and the passed sequence will be BLASTed against the genotype database to yield a result, which will be returned to the webpage through the PHP program (Figures 1A,B). Through the above procedures, the genotyping module of PmGT (http://vetinfo.hzau.edu.cn/PmGT) was developed (Figure 1).

Currently, PmGT provides the above services includes five menus: (1) the “Home” page gives a brief introduction of *P. multocida* etiological characteristics to help the users understand the bacterium; (2) the “Isolates” page displays the genotypes of *P. multocida* strains based on their whole genome sequences that are publicly available in NCBI; this page also provides the link for the users to download the genomes of these *P. multocida* strains from NCBI; (3) the “Genotyping” page enables the users to determine whether a putative isolate is a *P. multocida* and genotype *P. multocida* strains by using the whole genome sequence assembled from the sequencing reads (Figure 1C); (4) the “About” page summarizes the guidelines for the use of this web tool; (5) the “Contact” page provides the contact information of the developers.

### PmGT Shows the Same Accuracy With PCR Methods in Genotyping *P. multocida* Strains

To test the accuracy of PmGT, we used two methods to type 52 *P. multocida* isolates (HB01, HB02, HB03, HN04, HN05, HN06, HN07, HNA01~HNA22, HND01~HND21, HNF01, and HNF02) from our laboratory collection (27). First, we submitted their whole genome sequences to PmGT for genotyping. As a comparison, we also determined the capsular genotypes, LPS genotypes, sequence types, as well as the profile of the abovementioned 23-kinds of virulence genes by using PCR assays. All these 52 strains were genotyped by PmGT and through this online genotyping platform (Table 1). Genotyping by PCR assays confirmed these capsular, LPS, and MLST genotypes. PCR results of capsular and LPS genotypes are provided in Supplementary Figures S1, S2.

**Table 1:** Genotypes of 52 *Pasteurella multocida* strains determined via the PmGT Platform.

**Strain**	**Capsular genotype**	**LPS genotype**	**MLST genotype (Sequence type)**	**GenBank accession numbers**
HB01	A	L3	ST1	CP006976
HB02	A	L1	ST128	LYOX00000000
HB03	A	L3	ST3	CP003328
HN04	B	L2	ST44	PPVE00000000
HN05	D	L6	ST11	PPVF00000000
HN06	D	L6	ST11	CP003313
HN07	F	L3	ST12	CP007040
HNA01	A	L3	ST133	PPVG00000000
HNA02	A	L6	ST10	PPVH00000000
HNA03	A	L3	ST3	PPVI00000000
HNA04	A	L6	ST10	PPVJ00000000
HNA05	A	L6	ST10	PPVK00000000
HNA06	A	L6	ST10	PPVL00000000
HNA07	A	L6	ST10	PPVM00000000
HNA08	A	L3	ST3	PPVN00000000
HNA09	A	L3	ST3	PPVO00000000
HNA10	A	L6	ST10	PPVP00000000
HNA11	A	L6	ST10	PPVQ00000000
HNA12	A	L6	ST10	PPVR00000000
HNA13	A	L3	ST3	PPVS00000000
HNA14	A	L3	ST3	PPVT00000000
HNA15	A	L3	ST3	PPVU00000000
HNA16	A	L6	ST10	PPVV00000000
HNA17	A	L3	ST3	PPVW00000000
HNA18	A	L3	ST3	PPVX00000000
HNA19	A	L3	ST3	PPVY00000000
HNA20	A	L3	ST3	PPVZ00000000
HNA21	A	L6	ST10	PPWA00000000
HNA22	A	L6	ST10	PPWB00000000
HND01	D	L6	ST11	PPWC00000000
HND02	D	L6	ST134	PPWD00000000
HND03	D	L6	ST11	PPWE00000000
HND04	D	L6	ST11	PPWF00000000
HND05	D	L6	ST11	PPWG00000000
HND06	D	L6	ST11	PPWH00000000
HND07	D	L6	ST11	PPWI00000000
HND08	D	L6	ST11	PPWJ00000000
HND09	D	L6	ST11	PPWK00000000
HND10	D	L6	ST11	PPWL00000000
HND11	D	L6	ST11	PPWN00000000
HND12	D	L6	ST134	PPWM00000000
HND13	D	L6	ST134	PPWO00000000
HND14	D	L6	ST11	PPWP00000000
HND15	D	L6	ST11	PPWQ00000000
HND16	D	L6	ST11	PPWR00000000
HND17	D	L6	ST11	PPWS00000000
HND18	D	L6	ST11	PPWT00000000
HND19	D	L6	ST11	PPWU00000000
HND20	D	L6	ST11	PPWV00000000
HND21	D	L6	ST11	PPWW00000000
HNF01	F	L3	ST12	PPWX00000000
HNF02	F	L3	ST12	PPWY00000000

**Figure 2:**
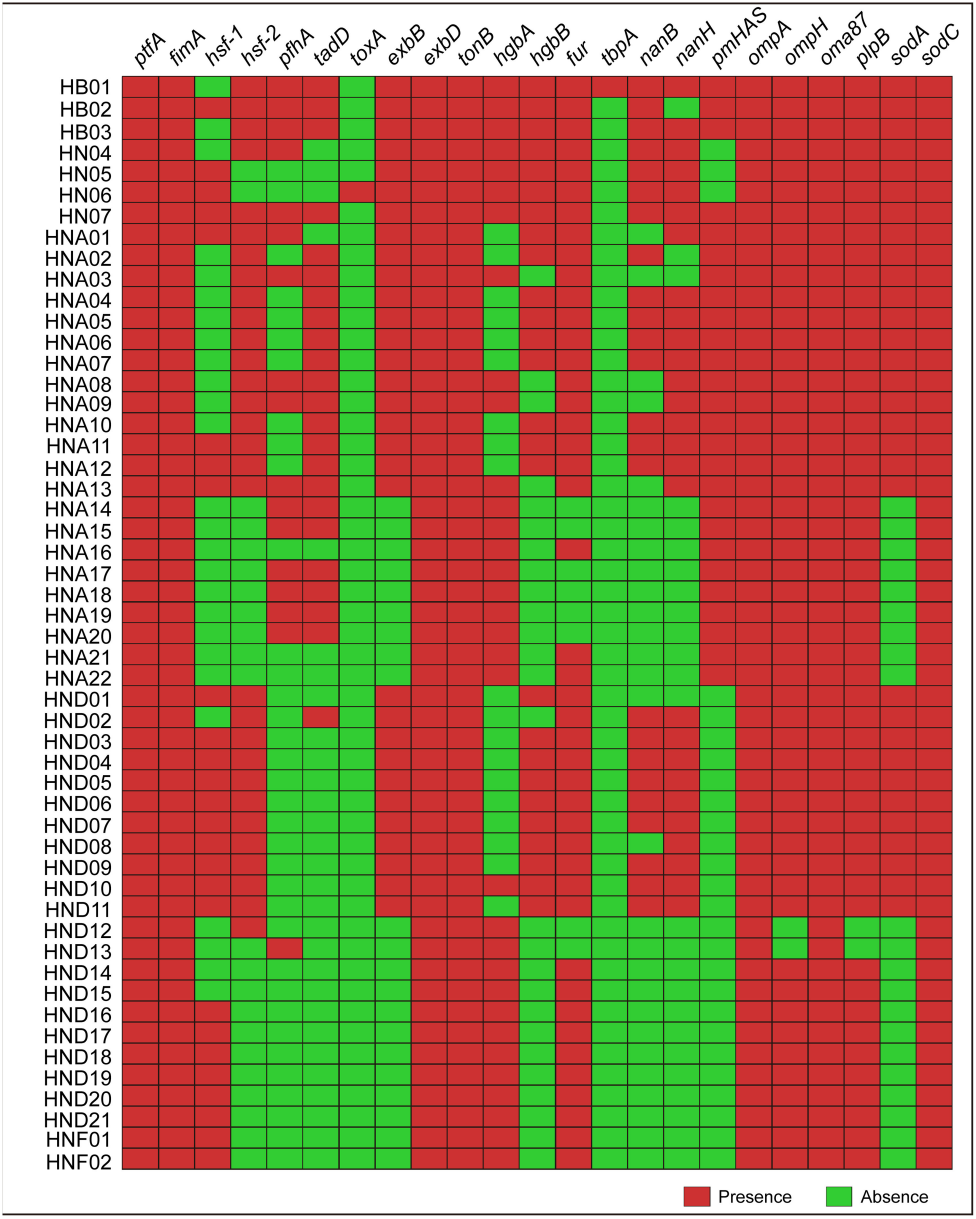
Heatmap showing the distribution of the 23 types of virulence genes (VFGs) among the 52 *P. multocida* strains from pigs. Boxes in red indicate a VFG is presence in the strain while boxes in green represent a VFG is missing in the strain.

Determination of the 23 types of virulence genes for each of the 52 strains by using this online system revealed that several genes (*ptfA, fimA, oma87*, and *sodC)* were broadly presented in the genome sequences genotyped (Figure 2). However, several genes (*hsf-1, hsf-2, pfhA*, and *tadD)* were heterogeneously distributed, and in particularly, none of the 52 sequences genotyped carried the *toxA* or *tbpA* genes (Figure 2). These results were also confirmed by PCR assays (Supplementary Table S1).

### Genotypes of *P. multocida* From Different Hosts

**Figure 3:**
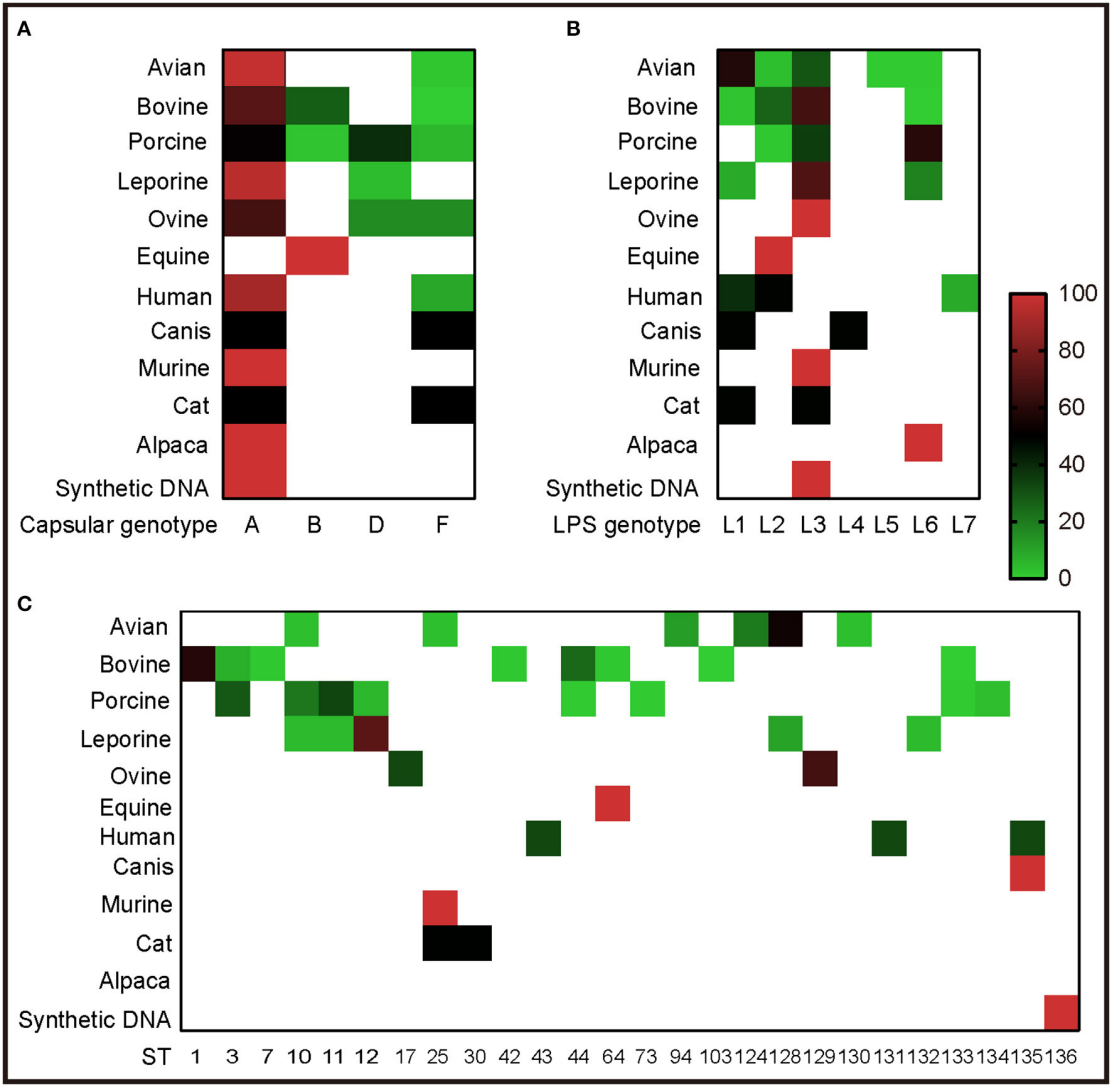
Heatmap revealing the association between capsular/LPS/MLST genotypes and *P. multocida* strains from different host species determined by PmGT. **(A)** Heatmap revealing the association between capsular genotypes and *P. multocida* strains from different host species; **(B)** Heatmap revealing the association between LPS genotypes and *P. multocida* strains from different host species; **(C)** Heatmap revealing the association between MLST genotypes and *P. multocida* strains from different host species. Percentages of sequences typed are shown with different colors displayed at right corner.

**Figure 4:**
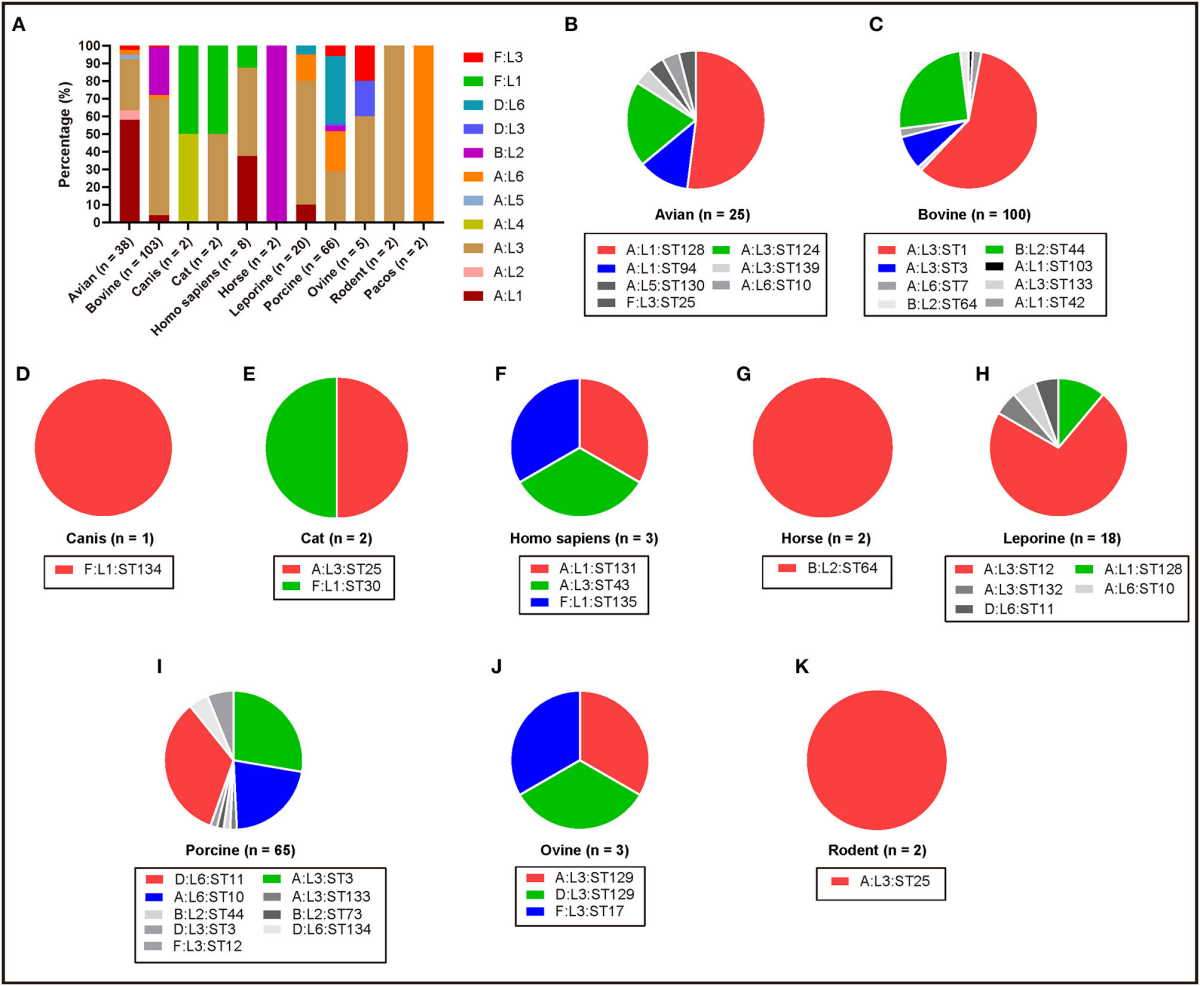
Column and pie charts showing the distribution of capsular: LPS genotypes and/or the capsular: LPS: MLST genotypes of *P. multocida* strains from different host species determined by PmGT by using the whole genome sequences. **(A)** Column chart showing the distribution of capsular: LPS genotypes of *P. multocida* strains from different host species; **(B–K)** Pie charts showing the distribution of capsular: LPS: MLST genotypes of *P. multocida* strains from avian species, bovine species, canis, cats, humans, horses, leporine species, pigs, ovine species, and rodents, respectively.

To understand the genotypes of *P. multocida* strains circulation in different host species, the 262 whole genome sequences of *P. multocida* strains were genotyped by PmGT. The results revealed that *P. multocida* isolates from different hosts displayed a certain preference for “capsular/LPS/MLST genotypes” (Figure 3). For example, most of the porcine strains were determined as capsular genotypes A (52%) and D (39%), LPS genotypes L3 (36%) and L6 (61%), sequence types ST3 (29%), ST11 (22%), and ST10 (34%), respectively; while most of the genotyped bovine strains were determined as capsular genotypes A (72%) and B (28%), LPS genotypes L3 (67%) and L2 (27%), and sequence types ST1 (59%) and ST44 (25%), respectively (Figure 3). When combining the capsular genotypes and the LPS genotypes, it revealed that most of the genotyped avian *P. multocida* were typed as A:L1 and A:L3, while most of the genotyped bovine *P. multocida* were typed as A:L3 and B:L2; the genotyped porcine *P. multocida* mainly belonged to D:L6, A:L3, and A:L6; while the genotyped leporine *P. multocida* mainly belonged to A:L3; most of the genotyped human *P. multocida* were typed as A:L3 and A:L1 (Figure 4A). If the capsular genotypes, LPS genotypes, and MLST genotypes were combined, most of the genotyped avian *P. multocida* were typed as A:L1:ST128, while most of the genotyped bovine *P. multocida* were typed as A:L3:ST1 and B:L2:ST44; the genotyped porcine *P. multocida* mainly belonged to D:L6:ST11, A:L3:ST3, and A:L6:ST10; while the genotyped leporine *P. multocida* mainly belonged to A:L3:ST12 (Figure 4).

**Figure 5:**
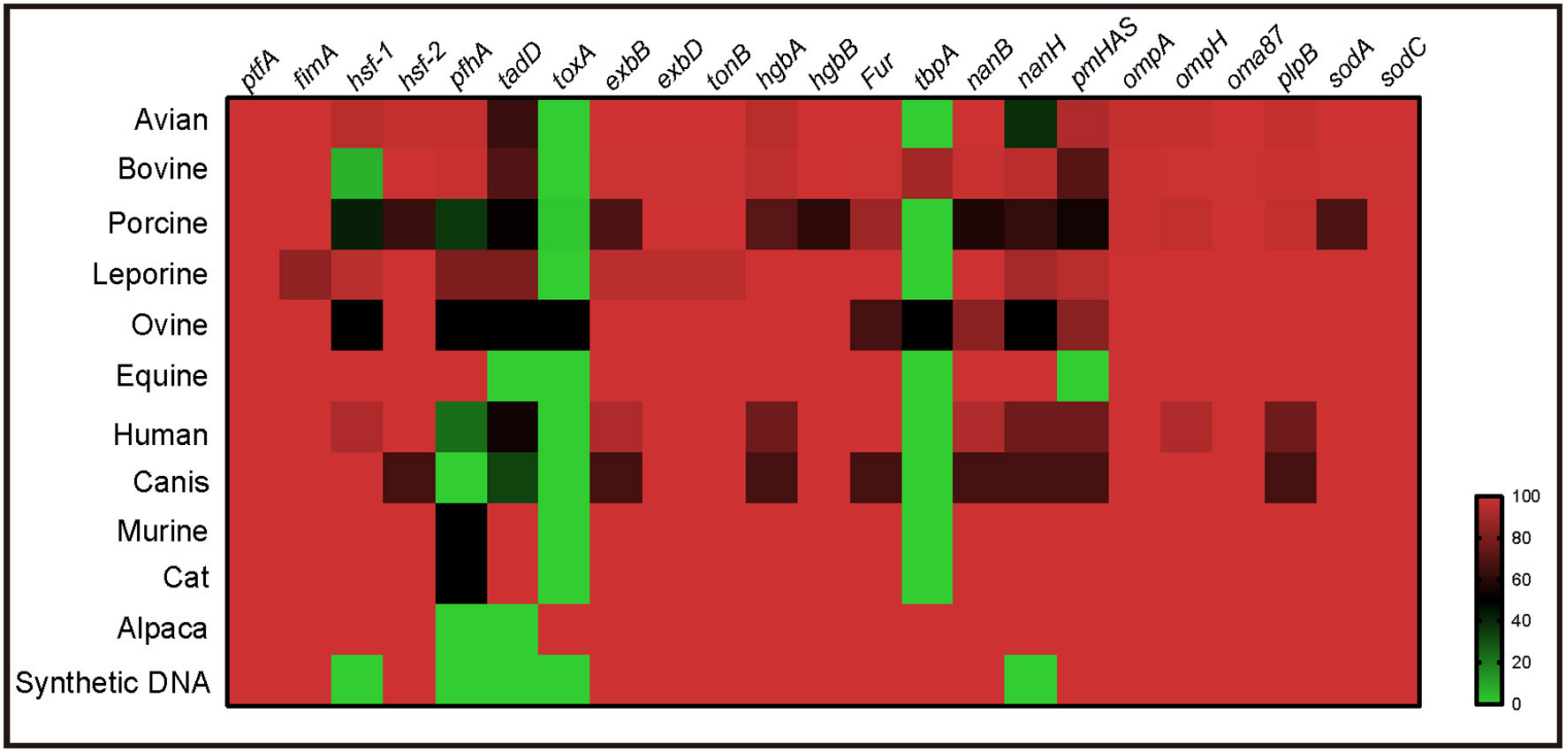
Heatmap revealing the association between virulence genes and *P. multocida* strains from different host species.

Virulence genotyping using the system developed herein revealed that the presence of multiple VFGs, including *ptfA, fimA, hsf-2, exbB, exbD, tonB, hgbA, hgbB, fur, nanB, nanH, ompA, ompH, oma87, plpB, sodA*, and *sodC*, was a broad characteristic of *P. multocida* strains from multiple host species (Figure 5). However, several VFGs were only determined in the genome sequences of *P. multocida* from certain hosts. For example, *toxA*, a gene encoding a dermonecrotic toxin, was found only in strains from pig, sheep, and alpacas, while *tbpA*, a transferrin binding protein coding gene, was found only in strains from cattle, sheep, and alpacas (Figure 5).

## Discussion

*P. multocida* is the causative agent of multiple diseases with a wide spectrum of host species, including humans and other primates (7–9). In addition, *P. multocida* isolates recovered from different hosts with different diseases can be classified in many different serovars/genotypes according to different typing systems (7, 9). Relying on only one or two typing systems is difficult to address the characteristics of *P. multocida* isolates from different host species and/or their association with different diseases. For example, *P. multocida* isolates from different host species might have the same capsular genotypes but possess different LPS genotypes and/or MLST genotypes; even those from different host species that share the same capsular, LPS, and MLST genotypes might carry different VFGs (27, 33). Therefore, we have proposed a combined “capsular: LPS: MLST” genotyping system that includes virulence genotyping to discriminate *P. multocida* isolates from different host species and/or those associated with different diseases (7). However, this combined genotyping system is multiplex PCR-based and is laborious and time-consuming.

Advances in bioinformatics and bioinformatical tools enable the application of whole genome sequence data for inclusion of various demographic information for bacterial characterization, such as capsular and LPS genotyping; the presence of adhesins, toxins, or other virulence factors (34). In the present study, we reported the development of a genotyping platform for distinguishing *P. multocida* isolates according to the bacterial whole genome sequences. Validation of the PmGT platform was performed on a collection of *P. multocida* isolates from our laboratory. Results revealed that this genotyping system provides consistent results of determining the capsular-, LPS-, MLST genotypes, and VFGs, as compared with that obtained using multiplex PCR-based typing systems. Compared to the multiplex PCR-based typing systems (18, 19, 21, 22) and traditional serological typing systems (13, 16), this genotyping system takes less time to yield results and does not require high-quality antisera, which represents a more efficient and cost-saving tool for characterizing *P. multocida* isolates in both epidemiological studies and clinical settings.

By using PmGT, the capsular-, LPS-, MLST genotypes, and VFGs of *P. multocida* strains from different hosts were determined according to the whole genome sequences. These results agree with those of the epidemiological studies (23, 24, 26, 35). For example, *P. multocida* serovars B: 2 and A: 3 strains are frequently associated with bovine haemorrhagic septicaemia and respiratory diseases, respectively (36, 37). It is known that *P. multocida* serogroups A and B are assigned to capsular genotypes A and B by multiplex PCR, respectively (18); while *P. multocida* Heddleston serovars 2 and 3 are assigned to LPS genotypes L2 and L3 by multiplex PCR, respectively (19). That is why the capsular: LPS genotypes of most of the bovine strains were determined as A: L3 and B: L2, respectively. In addition, *P. multocida* strains isolated from bovine haemorrhagic septicaemia are commonly determined as ST122 (38), this sequence type can be reassigned to ST44 by using the multihost MLST database (27). These findings could explain why *P. multocida* strains associated with bovine haemorrhagic septicaemia were typed as capsular: LPS: MLST genotype B: L2: ST44. Similar findings were also observed in *P. multocida* strains from the other host species. In particularly, most of the *P. multocida* strains from pigs were determined as capsular: LPS: MLST genotypes D: L6: ST11, A: L3: ST3, and A: L6: ST10. These results are also in agreement with the results of our previously epidemiological study (23), suggesting that these three genotypes, particularly genotype D/L6/ST11, are likely to be strongly associated with swine respiratory diseases. However, during our test we also found the capsular-, LPS-, and/or MLST-genotypes of several strains could not be determined by PmGT according to the whole genome sequences. After check the data we put forward several reasons to explain this result: (1) most of these non-typeable genomes are sequenced and assembled using the second-generation sequencing technologies and the quality of these genomes are not high, some of the genes used for capsular/LPS/MLST genotyping fell within the gaps between genome contigs in the assemblies (7); (2) the genome sequences might be those of the capsular nontypeable strains reported (23, 39); (3) several strains belong to novel sequence types and the current *Pasteurella multocida* MLST database do not include these sequence types.

In conclusion, we developed an online platform for *P. multocida* genotyping (PmGT platform), which combines whole genome sequence analysis tools with web 2.0 technologies. By using this system, we determined the genotypes of *P. multocida* isolates from different host species. Overall, this system represents a more convenient tool for *P. multocida* diagnosis in both epidemiological studies and clinical settings. More importantly, our study provides an example to develop rapid and efficient tools for bacterial diagnosis by using their whole genome sequences in the coming age of artificial intelligence.

## Data Availability Statement

The datasets presented in this study can be found in online repositories. The names of the repository/repositories and accession number(s) can be found below: https://www.ncbi.nlm.nih.gov/genbank/, MN938443-MN938455 and MT570166~MT570166.

## Author Contributions

ZP, BWi, JW, and BWu contributed to conception and design of the study. ZP, JL, WL, FW, LW, XW, and LH performed the experiments. ZP, JL, LW, and JW performed the statistical analysis. ZP wrote the first draft of the manuscript. ZP, HC, BWi, JW, and BWu revised the manuscript. All authors contributed to manuscript revision, read, and approved the submitted version.

## Funding

This work was supported in part by China Postdoctoral Science Foundation (grant number 2020T130232), the Fundamental Research Funds for the Central Universities of China (grant number 2662018JC034), Guangdong Provincial Key Laboratory of Livestock Disease Prevention (grant number YDWS1901), and Hubei Provincial Key Research & Development Program (grant number 2021BBA085).

## Conflict of Interest

The authors declare that the research was conducted in the absence of any commercial or financial relationships that could be construed as a potential conflict of interest.

## Publisher's Note

All claims expressed in this article are solely those of the authors and do not necessarily represent those of their affiliated organizations, or those of the publisher, the editors and the reviewers. Any product that may be evaluated in this article, or claim that may be made by its manufacturer, is not guaranteed or endorsed by the publisher.

## Supplementary Material

Supplementary Text 1 Nucleotide sequences and their GenBank accession numbers for the construction of a comparative database for *P. multocida* genotyping.

Supplementary Figure S1 PCR results of capsular genotypes of tested *P. multocida* strains in the present study.

Supplementary Figure S2 PCR results of LPS genotypes of tested *P. multocida* strains in the present study.

Supplementary Table S1 *Pasteurella multocida* genome sequences used in the present study.
