# Supplementary material for: Development of an Online Tool for Pasteurella multocida Genotyping and Genotypes of Pasteurella multocida From Different Hosts
Source: Front Vet Sci. 2021 Dec 17;8:771157. doi: 10.3389/fvets.2021.771157 (PMC8718711; doi:10.3389/fvets.2021.771157)
Supplement: Supplementary Text 1 — Nucleotide sequences and their GenBank accession numbers for the construction of a comparative database for P. multocida genotyping. [file Data_Sheet_1.ZIP › Supplementary materials/Figure S1.pdf]

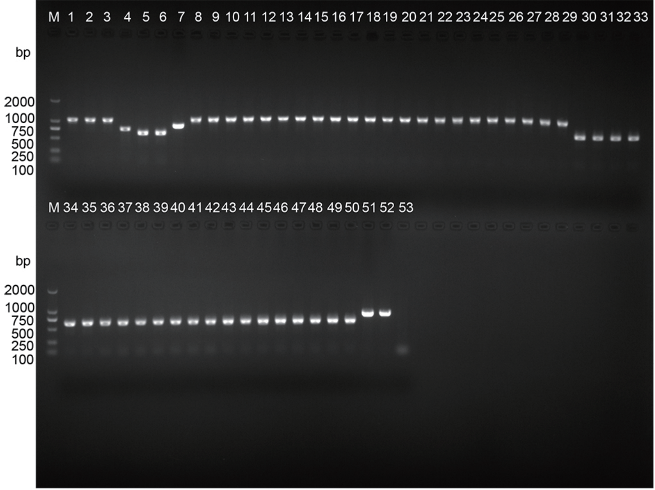

M: DL 2000 DNA marker
1: HB01 (capsular type A, 1048 bp)
2: HB02 (capsular type A, 1048 bp)
3: HB03 (capsular type A, 1048 bp)
4: HN04 (capsular type B, 758 bp)
5: HN05 (capsular type D, 647 bp)
6: HN06 (capsular type D, 647 bp)
7: HN07 (capsular type F, 852 bp)
8-29: HNA01~HNA22 (capsular type A, 1048 bp)
30-50: HND01~HND21 (capsular type D, 647 bp)
51-52: HNF01~HNF02 (capsular type F, 852 bp)
53: ddH2O control
