# Supplementary material for: Development of an Online Tool for Pasteurella multocida Genotyping and Genotypes of Pasteurella multocida From Different Hosts
Source: Front Vet Sci. 2021 Dec 17;8:771157. doi: 10.3389/fvets.2021.771157 (PMC8718711; doi:10.3389/fvets.2021.771157)
Supplement: Supplementary Text 1 — Nucleotide sequences and their GenBank accession numbers for the construction of a comparative database for P. multocida genotyping. [file Data_Sheet_1.ZIP › Supplementary materials/Figure S2.pdf]

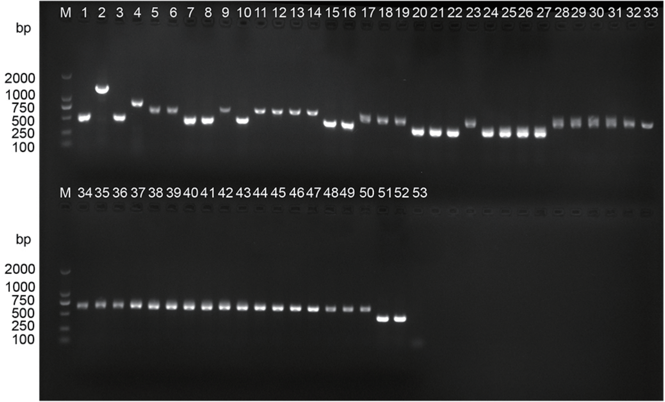

M: DL 2000 DNA marker

1: HB01 (LPS type L3, 474 bp)

2: HB02 (LPS type L1, 1307 bp)

3: HB03 (LPS type L3, 474 bp)

4: HN04 (LPS type L2, 810 bp)

5: HN05 (LPS type L6, 668 bp)

6: HN06 (LPS type L6, 668 bp)

7: HN07 (LPS type L3, 474 bp)

8: HNA01 (LPS type L3, 474 bp)

9: HNA02 (LPS type L6, 668 bp)

10: HNA03 (LPS type L3, 474 bp)

11~14: HNA04~NA07 (LPS type L6, 668 bp)

15~16: HNA08~HNA09 (LPS type L3, 474 bp)

17~19: HNA10~HNA12 (LPS type L6, 668 bp)

20~22: HNA13~HN15 (LPS type L3, 474 bp)

23: HNA16 (LPS type L6, 668 bp)

24~27: HNA17~HNA20 (LPS type L3, 474 bp)

28~29: HNA21~HNA22 (LPS type L6, 668 bp)

51-52: HNF01~HNF02 (LPS type L3, 474 bp)

30-50: HND01~HND21 (LPS type L6, 668 bp)

53: ddH2O control
